# Trophic interaction modifications: an empirical and theoretical framework

**DOI:** 10.1111/ele.12824

**Published:** 2017-09-17

**Authors:** J. Christopher D. Terry, Rebecca J. Morris, Michael B. Bonsall

**Affiliations:** ^1^ Department of Zoology University of Oxford Oxford OX1 3PS UK; ^2^ Biological Sciences, Faculty of Natural and Environmental Sciences University of Southampton Life Sciences Building 85 Highfield Campus Southampton SO17 1BJ UK; ^3^ St. Peter's College New Inn Hall Street Oxford OX1 2DL UK

**Keywords:** Food webs, indirect effects, interaction strength, mechanistic models, non‐trophic interaction, population dynamics, trait‐mediated indirect interaction, trophic interaction modification, trophic interactions

## Abstract

Consumer–resource interactions are often influenced by other species in the community. At present these ‘trophic interaction modifications’ are rarely included in ecological models despite demonstrations that they can drive system dynamics. Here, we advocate and extend an approach that has the potential to unite and represent this key group of non‐trophic interactions by emphasising the change to trophic interactions induced by modifying species. We highlight the opportunities this approach brings in comparison to frameworks that coerce trophic interaction modifications into pairwise relationships. To establish common frames of reference and explore the value of the approach, we set out a range of metrics for the ‘strength’ of an interaction modification which incorporate increasing levels of contextual information about the system. Through demonstrations in three‐species model systems, we establish that these metrics capture complimentary aspects of interaction modifications. We show how the approach can be used in a range of empirical contexts; we identify as specific gaps in current understanding experiments with multiple levels of modifier species and the distributions of modifications in networks. The trophic interaction modification approach we propose can motivate and unite empirical and theoretical studies of system dynamics, providing a route to confront ecological complexity.

## Introduction

Trophic interactions between species are often affected by other species within the community (Abrams 1983; Werner & Peacor 2003). There have been growing calls to incorporate these processes into studies of system dynamics (Bolker *et al*. 2003; Ings *et al*. 2009; Fontaine *et al*. 2011; Kéfi *et al*. 2012; Ohgushi *et al*. 2012; Sanders *et al*. 2014) since these trophic interaction modifications, hereafter ‘TIMs’ (Wootton 1993; Golubski & Abrams 2011), are often identified as the cause of unexpected responses to perturbations (Doak *et al*. 2008; Tack *et al*. 2011; Barbosa *et al*. 2017). Focussed, short‐term, studies with small numbers of species have repeatedly demonstrated that TIMs have the capacity to drive population dynamics (Werner & Peacor 2003; Preisser *et al*. 2005) and there is growing impetus to test the potential role of modifications in more complex systems. A greater understanding of how they influence population dynamics will be a key part of improving our ability to forecast how ecosystems will respond to change (Kéfi *et al*. 2012).

A very wide range of ecological processes can cause TIMs and historically these have been studied independently within ecological sub‐disciplines. While this breadth highlights the importance of interaction modifications and associated indirect effects, it may result in generalisations being missed and lost opportunities to draw inferences. Consistent and comparable quantification of TIMs that can be broadly applicable will be an essential tool to draw general conclusions regarding their impact (Abrams 1992, 2007; Okuyama & Bolker 2012). Although it can be comparatively clear which species are linked by trophic interactions, interaction modifications must be inferred from their effect upon the consumer and resource species. Furthermore, any species could hypothetically affect almost any interaction in a community to some degree. This potentially overwhelming complexity places great value on the ability to define when interaction modifications are ‘large’ enough to require consideration in order to understand the dynamics of a given system.

Here, we advocate extending our understanding of interaction modification effects beyond pairwise approaches through the use of an explicitly multi‐species TIM approach. To establish common frames of reference and explore the value of this approach, we develop a set of metrics to show how TIMs can be interpreted, quantified and linked to experimental data. We show that this TIM concept offers a lens to unite a range of important processes that has the potential to greatly enhance our understanding of these effects on both theoretical and empirical system dynamics.

### Delineating trophic interaction modifications

Trophic interaction modifications are defined as the modification of a consumer–resource interaction by a third species (Wootton 1993; Golubski & Abrams 2011). This common property can link a wide range of important ecological phenomena as modifications of functional responses and as such form a useful and discrete subset of the wider field of ‘non‐trophic interactions’ (Kéfi *et al*. 2012). Examples of sources of TIMs include foraging choices (Abrams 2010a) and associational defences (Barbosa *et al*. 2009), which act to cause non‐trophic interactions such as ecosystem engineering impacts (Sanders *et al*. 2014), fear effects (Brown *et al*. 1999) and mutualistic relationships (Holland *et al*. 2005). By focusing on effects that involve consumption, and not attempting to also include other processes that have been grouped into the category of ‘non‐trophic effects’ (such as reproductive interference, migration effects and mutualistic impacts on survival), we believe this TIM framework strikes a balance between grouping a wide range of ecological effects while still being able to examine common dynamic consequences in depth. Nonetheless many of the concepts discussed in this paper could also apply to non‐trophic interaction modifications, such as modulation of competition for space by a third species.

**Figure 1:**
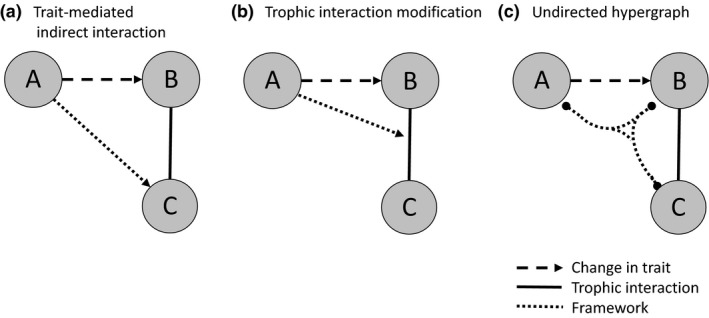
Illustration of the distinction between different frameworks that can represent the impact of the influence of third species on trophic interactions. A trait‐mediated indirect interaction approach (a) represents the resultant link between A and C, a trophic interaction modification approach (b) represents the change in the interaction strength, while an undirected hypergraph approach (c) represents a relationship between all three species.

Two alternate concepts for the study of this same set of ecological phenomena have developed (Table 1), which we will refer to here as the TIM approach and the trait‐mediated indirect interaction (TMII) approach, where ‘trait’ is broadly defined to include any property of an organism that affects its functional response or that of its consumer (Bolker *et al*. 2003). The TIM approach conceptualises the modification of the interaction as a distinct entity of study in itself, while the TMII approach emphasises the pairwise consequences of the modification. The distinction between proximate cause (change in trait), dynamic (change to the interaction) and consequence (resultant effect between species not otherwise linked) can be blurred and the terms conflated since the TIM and TMII approaches attempt to represent the same underlying ecology (Fig. 1).

**Table 1:** Terminology used to describe approaches focussing on pairwise effects and changes to interactions. Note that ‘higher‐order interaction’ has been used for both concepts

Process	Terminology	References
Pairwise effect	Trait‐mediated indirect interaction	Werner & Peacor (2003); Abrams (2007)
	Functional indirect interaction	Janssen *et al*. (1998)
	Trait‐mediated biotic indirect effect	Goudard & Loreau (2012)
	Trait‐initiated indirect effect	Abrams (2004)
	Trait‐transmitted indirect effects	Abrams (1995)
	Behavioural indirect effect	Miller & Kerfoot (1987)
	Chemical response indirect effect	Miller & Kerfoot (1987)
	Non‐consumptive predator effect	Preisser & Bolnick (2008)
	Non‐trophic interaction	Kéfi *et al*. (2015)
	Risk effect	Creel & Christianson (2008)
	Emergent multi‐predator effects	Sih *et al*. (1998)
	Higher‐order interaction	Vandermeer (1969)
Change to interaction	Trophic interaction modification	Golubski & Abrams (2011)
	Interaction modification	Wootton (1993)
	Rheagogy	Arditi *et al*. (2005)
	Environment‐mediated interaction modification	Wootton (2002)
	Associational resistance/susceptibility	Barbosa *et al*. (2009)
	Resource choice	Abrams (2010b)
	Prey switching	Koen‐Alonso (2007)
	Higher‐order interaction	Billick & Case (1994)

A pairwise TMII approach brings certain advantages; for example, facilitating direct comparisons with other classes of trophic and non‐trophic interactions through established network metrics and extensions into multi‐layer networks (Kéfi *et al*. 2016). However, this coercion of a process involving at least three species into an interaction between two species risks obscuring fundamental features and mechanisms that differentiate these trait‐mediated processes from other interactions (Golubski *et al*. 2016). As a very direct example, if species B of the system depicted in Fig. 1(a) is no longer present, there should no longer be an A–C interaction, but the dependence on B is not included in the specification of the A–C indirect pairwise link. In contrast, the TIM concept does not allow conventional network analysis (Newman 2010), since that requires a distinct set of nodes and links (edges). However, for this very reason, a TIM framework is a more direct representation of the process from a system dynamics perspective, in particular identifying the distinctive roles of the species involved.

A third approach using the mathematical concept of ‘hypergraphs’ has recently been proposed by Golubski *et al*. (2016). This framework represents interactions with ‘hyperedges’ that can link any number of species, in contrast to conventional network links that each represent interactions between exactly two species. This incorporates the multi‐species nature of effects caused by interaction modifications and allows the application of hyper‐dimensional extensions of network metrics. However, as Golubski *et al*. (2016) discuss, this approach is limited in its ability to represent the underlying dynamics as it does not yet allow the specification of the identity of modifier and interactors, or the directionality of the effect.

## Opportunities of TIM Approach

Compared to pairwise TMII analyses, a TIM framework provides a practical approach to consider a greater range of dynamic consequences from the same underlying ecology. TIMs provide a mechanism to translate potentially disparate and complex underlying processes into an understanding of how they lead to consequences at higher levels of organisation. To examine some of these opportunities, we first consider how the ‘strength’ of TIM effects can be defined. Here we outline five aspects of interaction modifications that can be considered in metrics of their magnitude to demonstrate the potential utility of the approach.

Firstly, the impact of an interaction modification can depend on the strength of the interaction being modified – a large proportional effect on a weak interaction may be less likely to have a large effect on the system as a whole. However, interaction strength has been specified by a host of different complimentary approaches (Laska & Wootton 1998; Berlow *et al*. 2004), ranging from consumption rate to response ratios. For simplicity, most simulation models seeking to evaluate the impact of TIMs specify them as relative modifiers of interactions (Křivan & Schmitz 2004; Arditi *et al*. 2005; Goudard & Loreau 2008). With the ability to consider explicitly the aspect of interaction strength that is being modified, the dynamic impact of the modification under consideration can become clearer.

**Figure 2:**
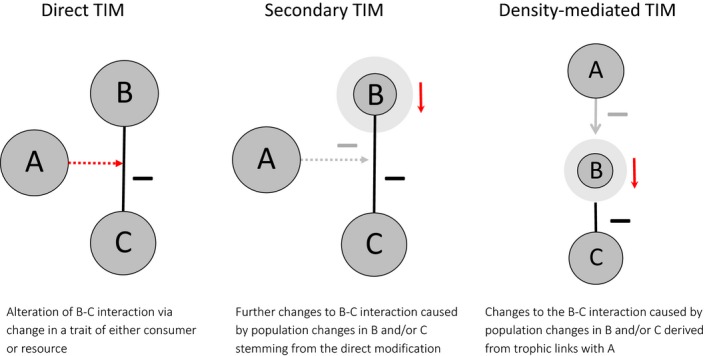
Illustration of distinction between direct and indirect trophic interaction modifications (TIM). The red arrows show the proximate driver of the change to the interaction. For the indirect cases, the grey arrows depict the process by which the interaction was modified.

Secondly, conceptions of TIM strength can consider the extent to which they incorporate indirect effects of TIMs and resultant feedbacks, with important consequences for the interpretation of experiments. The presence of a modifying species can change an interaction in three distinct ways, which we define as direct, secondary and density‐mediated TIMs (Fig. 2). As an example, consider a three‐level (plant–herbivore–predator) food chain where higher densities of the predator cause the herbivore to reduce its consumption rate of the plant. The TIM would reduce the herbivore–plant interaction directly, but the interaction would also be affected by the reduced herbivore population due to reduced food intake (secondary TIM) and because of predation losses (density‐mediated TIM). There is no guarantee that an indirect TIM would be in the same direction as, or of lesser magnitude than, the direct TIM. Many verbal descriptions of interaction modifications include only direct TIMs and their effects, yet where populations vary secondary and density‐mediated mechanisms can play considerable roles in experimentally observed changes in interactions. A TIM framework allows a more precise description of the scope of processes that are being described.

Thirdly, TIMs are inherently dynamic concepts and the full range of their impacts often only become visible in non‐equilibrial systems or after perturbations (Doak *et al*. 2008). If the modifying species is at low or unvarying density there may be little observable modification effect, despite a large potential to affect an interaction if the modifier's population was perturbed. Modifications can obscure their own impact on dynamics if they lead to the stabilisation of systems (and hence reduce the variation in interaction strength). There is therefore an important bi‐directional link between system dynamics and TIM strength, which pairwise approaches are unable to handle as easily.

Fourthly, modifications to interactions may occur only after a considerable time lag to changes in the modifier population (Bolker *et al*. 2003) and such delays can have notable effects (Hastings *et al*. 2007). Delays could stem from the time taken to learn behavioural responses, time for genetic selection to occur, or time taken for environmental changes to the ecosystem to develop. Multi‐scale models can incorporate delayed interaction modifications directly with a time lag, or more commonly with an additional state variable representing an environmental cue or a trait. The trait changes can be determined by adaptive considerations (Kondoh 2003; Valdovinos *et al*. 2010) or as a direct function of the modifier (Garay‐Narváez & Ramos‐Jiliberto 2009). A dynamic‐focused TIM approach is amenable to studying system responses over different timescales. Transient dynamic metrics can also be applied to investigate dynamics distinct to long‐term asymptotic behaviour. However, for the remainder of this paper, we will focus on the effect of changes that can be reasonably be modelled as instantaneous.

**Figure 3:**
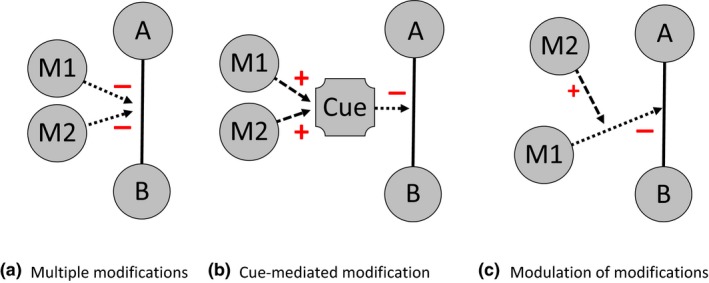
Illustration of how multiple modifying species can act on an interaction. Dotted lines indicate interaction modifications; dashed lines indicate other processes related to interaction modifications: increase in ‘cue’ in (b) and a modification of a modification in (c).

Finally, multiple modifying species can exert simultaneous modifications upon a focal interaction (Fig. 3a, Relyea 2003; Golubski & Abrams 2011). While it is possible to partition consumption directly to particular species, partitioning effects of modifications poses a fundamentally challenging question since the process is not directly observable. Multiple modifying species can cause changes in the same, opposite or a completely different direction in the trait‐space of the interactors‐alternatively described as functionally equivalent, functionally inverse or functionally diverse (Herzog & Laforsch 2013). By focussing on the combined changes to interactions, the TIM framework aids the generation of hypotheses for how modification effects combine (Golubski & Abrams 2011) and a route to possible additional model structures, such as introducing cue variables (Fig. 3b, Garay‐Narváez & Ramos‐Jiliberto 2009). Furthermore, the TIM framework allows a distinction between multiple modifications acting simultaneously and the modulation of modifications where a TIM is itself directly modified by a fourth species (Fig. 3c; for e.g. see Liere & Larsen 2010).

## Metrics

Concepts of TIM strength are contingent on the extent to which information about the wider system is incorporated. Table 2 outlines a set of TIM metrics, both long‐standing and newly proposed, that sequentially increase the amount of information incorporated about the system. TIM effects have been studied in a wide range of theoretical frameworks (Peacor & Cressler 2012) including: network re‐wiring (Staniczenko *et al*. 2010), sign‐only matrices (Dambacher & Ramos‐Jiliberto 2007), fitness‐based models (Valdovinos *et al*. 2010), explicit cue‐state variables (Garay‐Narváez & Ramos‐Jiliberto 2009) and individual‐based models (Peacor *et al*. 2007; Railsback & Harvey 2013). Here, we focus on a common framework where sets of differential equations represent the rate of each population's density change and the TIM is represented by additional functional response components (Arditi *et al*. 2005; van Veen *et al*. 2005; Holt & Barfield 2013). This population‐level representation abstracts over individual variation and the underlying (often trait‐based) causes of interaction modifications to focus on resultant effects at the level of the species. Nevertheless, the distinctions between TIM effects as measured by different metrics are not specific to this modelling approach.

**Table 2:** Metrics of trophic interaction modification strength, discussed in more detail in the main text. Indices: *i* = prey, *j* = predator, *k* = modifier species

Metric	Composition	Explanation
Modification parameter	$c_{ijk}$	Parameter in function that links modifier species density to consumption rate in the functional response model (van Veen *et al*. 2005)
Modification term	$f(c_{ijk},k)$	The term by which a functional response parameter, such as attack rate, is modified. It incorporates modifier density and TIM model structure (Golubski & Abrams 2011)
Flux change	$\Delta_{rel}=\frac{f(i,j,k_{=0})}{f(i,j,k)}$	The difference in the interaction strength (as measured by biomass or energy flux) due to the modifier, as either a raw difference or a ratio (Peacor & Werner 2004)
	$\Delta_{abs}=f\left(i,j,k_{=0}\right)-f\left(i,j,k\right)$	
Change in $B_{CR}$	$\frac{I^{*}}{I_{c_{ijk}=0}^{*}}$	The relative change in biomass potential of the resource ($B_{CR}$, computed from the relative change in equilibrium density of the resource in the presence of the consumer, Gilbert *et al*. 2014) due to a TIM as the ratio of the equilibrium value of the prey with and without the TIM
Coefficient of variation in modification	$\frac{\sigma_{f\left(k\right)}}{\mu_{f\left(k\right)}}$	For non‐stationary systems, the ratio of the standard deviation of the interaction strength modification divided by the mean modification over a period of time
Elements of Jacobian matrix	$\frac{\partial \dot{I}}{\partial K},\frac{\partial \dot{J}}{\partial K}$	TMII framework metric representing the direct effects of the modifier species on each interactor (Abrams 2008; Okuyama & Bolker 2012)
Partial derivatives of Jacobian matrix	$\frac{\partial }{\partial K}A_{ij},\frac{\partial }{\partial K}A_{ji}$	The change in direct interaction strengths between the interactors with respect to modifier density
Partial derivatives of inverse negative Jacobian matrix	$\frac{\partial }{\partial K}{\left(-A^{-1}\right)}_{ij},\frac{\partial }{\partial K}{\left(-A^{-1}\right)}_{ji}$	The change in total (indirect and direct) interaction strength between the interactors with respect to modifier density

### Modification parameter

With a functional response model that includes a TIM it is possible to identify a parameter that specifies the strength and direction of the TIM influence on the interaction. These values can be found by fitting models to experimental data (van Veen *et al*. 2005) and can be specified directly in models (Křivan & Schmitz 2004; Goudard & Loreau 2008; Holt & Barfield 2013), thereby providing a direct link between experiment and theory. A range of symbols have been used for the TIM parameter in the literature, here we use $c_{ijk}$ to represent the effect species *k* has on the consumption of species *i* by species *j*. However, exactly how the resultant modification relates to this parameter is critically dependent on the form of the functional response model used. While some studies have used fully linear representations of TIMs (e.g. Bairey *et al*. 2016), non‐linearities in functional responses and the modification effect are likely to be prominent. It is possible for any aspect of a non‐linear functional response to be modified (Preisser & Bolnick 2008; Kéfi *et al*. 2012) including consumption rate (Arditi *et al*. 2005; Goudard & Loreau 2008), handling time (Vos *et al*. 2004), sigmoidality (Alexander *et al*. 2013) or additional interference terms (Vos *et al*. 2001; van Veen *et al*. 2005; Larsen 2012).

Given the huge diversity of possible models, it is useful to consider separately the underlying functional response model, the choice of the affected parameter, and the sub‐model linking the TIM parameter to changes of the affected functional response parameter. Previous work has used a wide variety of functions to link the density of the modifier to the modification term, outlined in Appendix 1 in Supporting Information, to introduce a variety of thresholds and non‐linear relationships. So far, there has been little theoretical comparison of different generic TIM functional responses (although see Holt & Barfield 2013). With the exception of prey switching effects (Koen‐Alonso 2007; Abrams 2010b), the only class of TIM that are often implicitly included in population dynamic models, there is little experimental data to inform the choice of TIM functional form. Until a significant body of data develops, the choice of formulation will depend on fit to biologically informed criteria, such as those suggested by Goudard & Loreau (2008).

### Modification term

The term representing the effect of the TIM within the functional response model incorporates the density of the modifier species. This gives a measure of the modification at a particular system state. As it is a sub‐element of the functional response model, the use of this term as a metric requires contextualisation within the wider model being used. The modification of different aspects of the functional response (such as handling time, attack rate or total consumption rate) can have very different effects on the change in consumption at different densities of interactors. This metric has had particular use in describing the combined effect of multiple modifications (Golubski & Abrams 2011).

### Change in flux

The most direct approach to incorporate the strength of the interaction being modified is to calculate the change in flux rate (biomass or energy) due to the modification. Either a ratio or an absolute flux difference can be considered. Indirect TIM effects (see Fig. 2) can lead to mixed responses to modifiers making it important to consider which effects are included in the measurement of flux change for comparisons to be meaningful.

### Change in *B*_*CR*_

The *B*_*CR*_ framework (Gilbert *et al*. 2014) proposes a metric of interaction strength that relates the energy flow between a consumer and a resource to static measures of the resource biomass in order to measure the biomass potential of the resource captured by the consumer. It can be calculated from the equilibrium biomass of the resource with and without the consumer being present, as well as directly from model parameters (Gilbert *et al*. 2014). As such it provides a way of linking empirical and theoretical studies and has been shown to be useful in relating interaction strength to stability (Nilsson & McCann 2016). Conveniently, as we show in Appendix 2 in Supporting Information, the relative change in this metric with and without the TIM can simplify to the relative density of the resource with and without the TIM. This is experimentally tractable compared to metrics that need to measure the flux and the metric can also be defined from model parameters (e.g. see Appendix 2 in Supporting Information).

### Coefficient of variation in modification parameter or interaction strength metric

Trophic interaction modifications are likely to be particularly critical in systems not at an equilibrium, but assessing the ‘strength’ of TIMs in such cases poses challenges. Where a model of the system is available, the coefficient of variation (the ratio of standard deviation to mean) of the modification parameter term can represent the dynamic changes a TIM is exerting. However, a low degree of variation in parameter modification does not necessarily mean the TIM is not having a significant impact since it is readily possible to show TIM effects that lead to the stabilisation of cycling systems (Appendix 3 in Supporting Information). Measures such as coefficient of variation in flux or other interaction strength metrics can also be derived from non‐equilibrium systems. These can be considered with reference to the level of environmental stochasticity underlying the system. There is considerable room within the TIM framework for a more sophisticated understanding of how modification effects interact with system variation to determine population dynamics (Peacor & Cressler 2012).

### Partial derivatives of Jacobian matrix

The metrics outlined above have represented a given TIM with a single value. An interaction is not necessarily symmetric in its effect on the interactors which can result in a pair of values for each TIM, representing the effect of the interaction modification on each species. Jacobian matrices of community dynamics models are composed of the partial derivatives of each species’ population change functions with respect to each species in the community. Within a pairwise TMII framework, the elements from this matrix have been put forward as a metric of modification effect (Abrams 1984). These correspond to the effect of modifier density on the modified species population growth. A development of this metric, taking the proportional change in growth rates with respect to the modifier, has also been put forward (Abrams 2008; Okuyama & Bolker 2012) to compare trait‐and density‐mediated indirect interactions at a particular community state. We propose here a related metric aligned with the TIM concept that is able to distinguish TIM effects from direct interactions linking the modifier and interactor. This can be computed by taking the partial derivative with respect to the modifying species of the Jacobian matrix elements that specify the modified interaction. This corresponds to the effect a modifier species has upon the direct interaction. For simple models it is possible to calculate interpretable expressions for how this metric responds to the system parameters. A significant feature of this metric is that it specifies the dynamic relationship of modifier and the interaction for a particular community at a given state, most likely an equilibrium. As with all Jacobian and gradient based metrics, it involves linearisation and hence is only strictly valid for system states very close to that in which it was defined, which could hinder direct experimental assessment.

### Partial derivatives of inverse negative Jacobian (‘net‐effects’) matrix

The previous metric can be extended to include indirect interactions between species. The inverse of the negative Jacobian matrix ($-A^{-1}$) represents the combined direct and indirect interactions between species (Levine 1976; Bender *et al*. 1984). Partial derivatives of elements of this ‘net‐effects’ matrix with respect to the modifier species density equate to the effect the modifier has on the total effect of the interactors on each other. Such an approach may lead to a more thorough understanding of the extent of impact that a TIM is having within a given system. However, there are some significant difficulties. Expressions of the value of this metric in terms of model parameters can be uninterpretably complex and numerical analysis can propagate errors due to the multiple differentiation and whole matrix manipulation steps. More fundamentally, because indirect effects are incorporated, values can be non‐zero when no explicit TIM effect is included due to density‐mediated TIM effects (see Fig. 2 and Case & Bender 1981).

### Comparison of metrics

**Figure 4:**
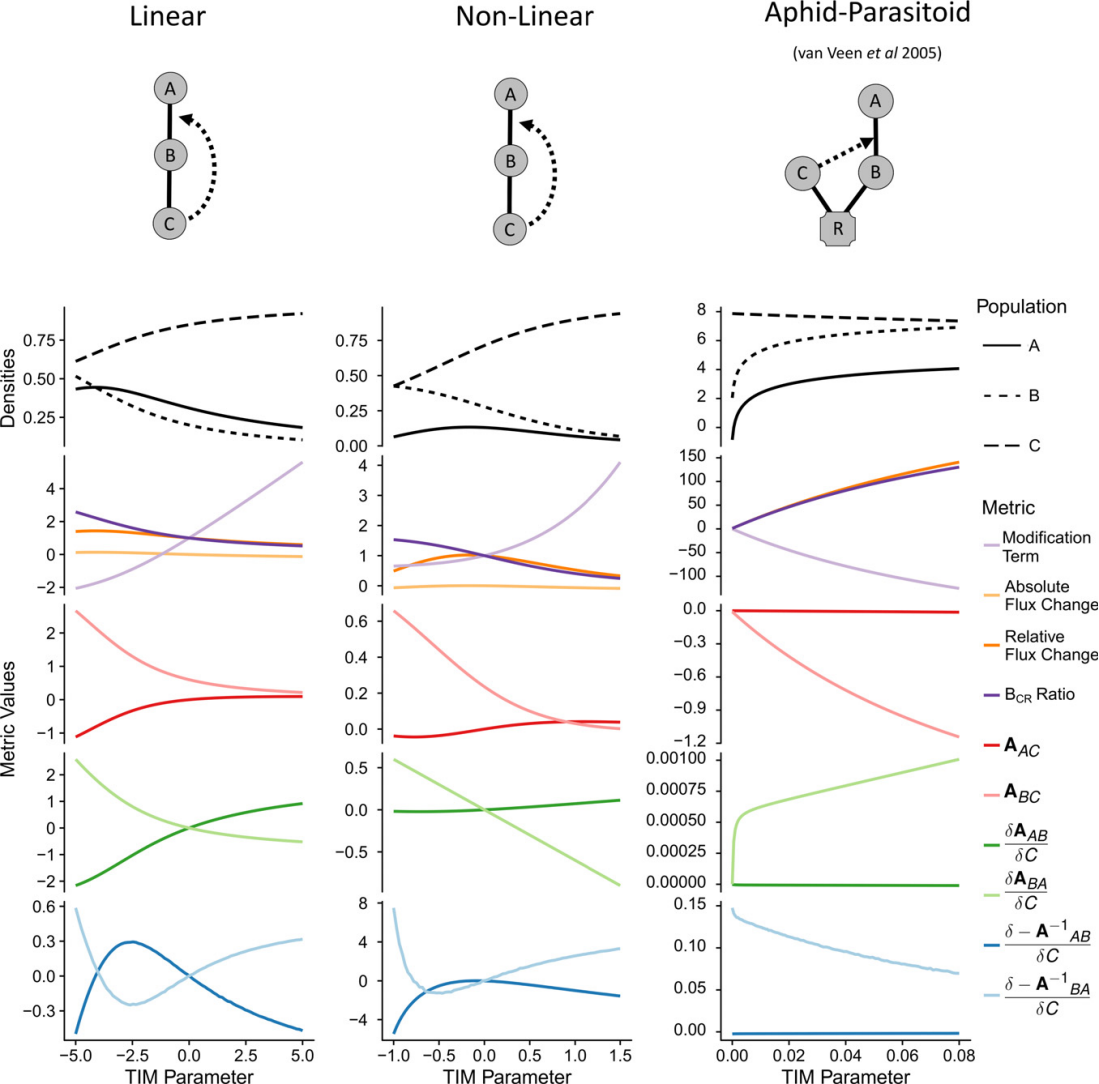
Demonstration that trophic interaction modifications (TIM) metrics display different qualitative and quantitative responses to changing the underlying parameter representing the TIM strength. Full model structures and parameters are given in supporting information. Note that the aphid‐parasitoid system population densities are on a log‐scale.

The need to differentiate between the diversity of potential approaches to TIMs is highlighted by a lack of consistency in the concepts of TIM strength. To demonstrate this, we applied the different metrics to three model systems with TIMs (Fig. 4): a tri‐trophic food chain with linear functional responses and TIM, a tri‐trophic system with non‐linear functional responses and TIM and the experimentally parameterised aphid‐parasitoid system described in van Veen *et al*. (2005). In each case, the strength of the model's TIM parameter was varied and the metric in question recalculated at the resultant equilibrium densities. Full model details are given in Appendix 4 in Supporting Information.

There are notable quantitative and qualitative differences in how the strength of the TIM, as measured by different metrics, responds to the changing underlying TIM parameter (Fig. 4). Since they measure different properties, it is not possible to propose one metric as universally superior. Successfully applying TIM strength metrics to specific questions will require measures of ‘strength’ that have tangible links to the theoretical concepts being considered, requiring a repertoire of diverse approaches. Furthermore, many approaches require specific conditions (such as the system being at equilibrium) that reduce their universality. Improving our understanding of how different aspects of interaction modifications relate to each other will be an important area for further research. While ‘lower‐level’ metrics are likely to be simpler to quantify, ‘higher‐level’ metrics place the modification in context and can provide more information on their role and impact in a dynamic system.

With the exception of the modification parameter, the metrics represent the strength of the TIM at a particular community state. These can provide considerable detail and insight about the process occurring in the system, but extending conclusions to consider larger perturbations may be challenging. This trade‐off reflects the highly dynamic nature of interaction modifications where taking a snapshot is essential to describe a system property concisely. For this reason, quantifying model parameters has particular advantages for understanding the system dynamics. Combined with a model specification they can describe the underlying process in a way that is not specific to a given system state and can allow the derivation of other measures.

Many of the apparently idiosyncratic relationships between the TIM parameter and the TIM strength observable in Fig. 4 are caused by interactions via changing equilibrium densities, with significant consequences for the interpretation of experimental data. Cascading effects of the changing link strength can counter the naive expectation that increasing the magnitude of the TIM parameter should lead to greater recorded ‘strength’ of the TIM. The capacity to explore the multi‐facetted nature of TIM effects is a significant advantage of this approach.

## Linking Theory and Experimental Systems

There is a significant gap between the concepts of interaction modifications that are used in theoretical or simulation studies and the objectives of the majority of experimental work. Despite repeated calls to try and close this gap (Bolker *et al*. 2003), a focussed programme of work has yet to emerge (Kéfi *et al*. 2012; Peacor & Cressler 2012). Key questions regarding the distribution, functional form and impact of TIMs in natural systems are only starting to be addressed (Perfecto *et al*. 2014; Kéfi *et al*. 2015; Suraci *et al*. 2016). The focus of a considerable number of experiments has been to identify the relative roles of trait‐mediated and density‐mediated effect pathways in tri‐trophic systems (Peacor & Werner 2004; Bolnick & Preisser 2005; Preisser *et al*. 2005). These studies have played a significant role in demonstrating that such effects can have major impacts, despite issues with the interpretation and duration dependence of such experiments (Abrams 2001, 2008; Okuyama & Bolker 2007). However, other experimental approaches will become necessary to deepen our understanding of these processes both to understand the causal mechanisms underlying TIMs and to quantify the modification.

A model‐fitting approach is able to overcome some of the challenges involved in quantifying modifications and their effects. Ideally, experiments seeking to investigate the effect a presumed TIM is having on a system would use an experimental treatment where the putative modifying species is present and trophically connected to the web, but the modifying effect is blocked. However, there are few systems where this is feasible. Consequently, many studies have conducted the reverse experiment, introducing a modification without the modifier being trophically connected to the system. A common experimental protocol has been to observe the effect of a single level of disabled, fear‐inducing, predator on the consumptive behaviour of another species (Werner & Peacor 2003). The extent to which these studies can inform our understanding of system dynamics is limited by the lack of information about the shape of the response and the lack of feedbacks to the modifier species (Bolker *et al*. 2003; Abrams 2008; McCoy *et al*. 2012; Okuyama & Bolker 2012). A shift in emphasis towards a TIM‐centred approach based on functional responses, as we outline here, rather than attempting to straightjacket such processes into an indirect‐interaction framework, will provide a profitable route of analysis. This would apply equally to modelling approaches at either individual or population levels.

### Short‐term experiments

**Figure 5:**
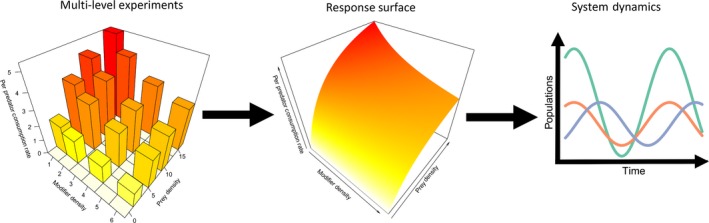
Illustration of how the results of multi‐level experiments can be used to specify a response surface that is of value for understanding system dynamics. Short‐term functional response experiments that include multiple levels of both prey and modifier can be used to define a function corresponding to a response surface, either by fitting parameters of a mechanistic model or with a regression based spline model. Such a function can then be used as part of models of the population dynamics of the system.

Ideally, experimental studies seeking to quantify TIMs would seek to parameterise a functional response model by fitting a response surface of the strength of the interaction across multiple levels of the modifier (Fig. 5; Denny & Benedetti‐Cecchi 2012; Okuyama & Bolker 2012). While such studies are inevitably more labour intensive than single‐level studies, Okuyama & Bolker (2012) present simulations that suggest even a low level of replication would provide useful results. Experimental functional responses have been fitted at different levels of modifier (e.g. Kehoe *et al*. 2016; Wasserman *et al*. 2016) and it would be a relatively small step for similar studies to define a single functional response model. TIMs themselves can be context dependent, for example, depending on prey hunger levels (Gravem & Morgan 2016) or temperature (Sentis *et al*. 2017). As it is well established that TIM effects have the potential to be large (Werner & Peacor 2003), future studies would be most valuable if they are as representative of natural conditions as possible rather than being designed with the aim of demonstrating an effect.

### Long‐term experiments

Model selection and parameter fitting with time series can give powerful insights into system dynamics (Mueller & Joshi 2000). Studying populations displaying natural dynamics can avoid the problems of contextualisation with short‐term experiments. Several studies have experimentally introduced a modification to a system, such as a predator cue, and studied the resulting dynamics (Peacor *et al*. 2012; Frago & Godfray 2014; Suraci *et al*. 2016). However, to the best of our knowledge, as yet only one study has included the modifier species treatment as a dynamic part of the system and gathered time series data to fit a mechanistic model (van Veen *et al*. 2005). Where the modifier is not a dynamic part of the system, cue‐based studies risk making an assessment of the effect of an altered parameter, rather than the effect of the dynamic nature of the TIM, as discussed further below.

Unfortunately, the difficulties commonly encountered when fitting models to time series could be exacerbated when studying interaction modifications. In order to detect changes to interactions the unmodified pairwise relationships must be well characterised. Furthermore, for robust model selection it is necessary for the populations to vary sufficiently for the system to traverse a significant part of the multi‐dimensional space specified by the modifiers and the interactors. In many systems, experimental perturbations would be necessary for this to occur in a reasonable period of time. Tight correlations between the modifier and either of the species involved in the interaction may hinder accurate parameterisation. Characterisation of the model structure is likely to have to remain the domain of short‐term studies since model selection in addition to model‐fitting is likely to require data beyond most available or feasible datasets. Because of the high level of experimental effort necessary, preliminary analysis using detailed models would be of value to examine the potential impacts of TIMs and aid the design of appropriate experiments.

Using time series methods to detect TIMs *de novo* is challenging because of the large potential number of modifications and hence a high risk of identifying artefacts. Nonetheless, it may be possible to removing split infintive calculate Jacobian‐based metrics directly from reconstructed manifolds based on long time series (Deyle *et al*. 2016). Well‐studied systems with existing time series also offer a wide scope to investigate TIM effects (Peckarsky *et al*. 2008) and would reduce the need to conduct new TIM‐specific time series experiments.

## Outlook

The stronger conception of interaction modifications we suggest here will allow new analyses and perspectives. One such area is highlighting TIMs that exert dynamic influences, compared to those emanating from species with comparatively fixed densities that may exert a strong, but unvarying influence. While stable equilibria may be a consequence of TIMs, modifications within systems at equilibrium introduce a constant change and the risk of examining the effect of a changed trait, not an additional dynamic property. For example, the stabilising effect of a TIM in three‐species model food chains (Křivan & Schmitz 2004; Holt & Barfield 2013) can be almost completely replicated by directly changing the model parameters to represent the modification without introducing a dynamic TIM (Appendix 5 in Supporting Information). It is important to consider the situations for which specifically introducing the added complexity of TIMs is valuable. For example, many plants have been shown to release volatiles to attract enemies of their herbivores (van Veen 2015). Where the trait modification is unrelated to the plant population density this could be represented by a statically changed functional response. This would be a more parsimonious representation of the process than introducing a dynamic TIM from the plant population to the herbivore‐enemy interaction. However, where the attractant effect is dependent on other herbivores (e.g. Vos *et al*. 2001) the TIM framework has much to offer by representing the relationship between the other herbivore and the enemy‐herbivore interaction.

This paper has largely discussed modelling at a population level, making the simplifying assumption that populations are homogenous and that their dynamics are tightly coupled. There is a body of evidence that intraspecies behavioural syndromes can exert a strong impact on ecological processes (Sih *et al*. 2012) and it has been demonstrated that some individuals consistently exhibit bolder behaviours in response to predation threat (Griffen *et al*. 2012). Even without a diversity of behavioural syndromes, environmental heterogeneities can create differences in how interactions of individuals are modified. For example, it has been shown that hunger level can determine whether individuals respond to predator cues by reducing their foraging (Gravem & Morgan 2016). One route to address the effects of intraspecific diversity (Bolnick *et al*. 2011) would be individual‐based modelling approaches (Peacor *et al*. 2007; Railsback & Harvey 2013). In principle, it would be possible to build up from individual measurements of response to modifiers to create individual‐based models (DeAngelis & Mooij 2005), although the demands on data would be high. Additionally, relatively abstract population‐level models cannot easily provide a causal explanation for the modification to interactions. ‘Bottom‐up’ approaches would supplement the population dynamic approach that has been principally discussed in this paper, in particular for their potential to show how individual behavioural optimisation in response to the trade‐offs involved in foraging choices leads to interaction modifications (Werner 1992).

While their ubiquity has been well established (Werner & Peacor 2003), the distribution of TIMs in empirical networks is a major unknown that will need to be addressed. There is every reason to expect that non‐random distributions will impact how TIMs influence community dynamics. Nonetheless, simulation studies using random networks of TIMs can still provide useful insights – much useful work was done with random trophic networks (May 1973; Allesina & Tang 2015) before and since detailed experimental data on trophic webs was developed. However, theoretical simulation studies must carefully consider the consequences of their choices of inter‐TIM relationships. If TIMs combine non‐additively, introducing a balanced distribution of positive and negative TIM parameters can shift average interaction strength, a key determinant of food‐web properties (Berlow *et al*. 2004). Goudard & Loreau (2008, 2012) suggest that such shifts in interaction strength may have contributed to the relationship between TIM connectance and ecosystem functioning they found in their simulation study. Calibrating modifications to maintain the average interaction strength may be challenging for complex communities. A profitable route may be to compare results to matched simulations that vary average interaction strength to identify where results can be attributed specifically to dynamic effects of TIMs.

There is currently only one study that attempts to represent the distribution of non‐trophic interactions in a whole natural community (Kéfi *et al*. 2015). It is highly unlikely that TIMs will be distributed randomly as current models (Arditi *et al*. 2005; Goudard & Loreau 2008; Lin & Sutherland 2014; Bairey *et al*. 2016) have assumed (Golubski & Abrams 2011). In particular, there is likely to be a tight relationship between the TIM topology and the underlying trophic network. For instance, it would be reasonable to expect that TIMs are more prevalent between species that are closely trophically connected. Despite the large challenges, there are reasons to be optimistic that significant progress can be made in empirically determining the distributions of TIMs in more systems. Mayfield & Stouffer (2017) demonstrate a model selection framework to identify TIM effects in a community of plants. There is also the opportunity to make use of allometric scaling relationships to make pattern‐based models for the distribution of modifications in a system (Krenek & Rudolf 2014).

## Conclusion

The field of non‐trophic interactions is poised to build upon demonstrations of possible effects towards an understanding of their role in the dynamics of natural systems. Trophic interaction modifications are a key set of processes where there is particular potential for progress. Theoretical work demonstrates that almost any set of dynamics can be generated from a model incorporating TIMs, necessitating communication between theory, laboratory experiment and field work to constrain possibilities (Bolker *et al*. 2003). Here we have put forward specific examples of the additional perspectives and depth that a TIM framework can bring and hope this encourages those planning experiments to take advantage of the opportunities. As with estimates of interaction strength (Laska & Wootton 1998), there is a benefit to identifying diverse perspectives for the strength of interaction modifications to understand and improve the links between theory and experiment. With a shift towards a focus on the modification itself as a discrete entity, we believe there are many exciting prospects to improve our understanding of ecosystem dynamics.

## Authorship

JCDT initiated the research, and JCDT, MBB and RJM contributed to the ideas presented in the manuscript. JCDT conducted the research, facilitated by discussions with RJM and MBB. JCDT wrote the first draft of the manuscript, and all authors contributed substantially to revisions.

## Data Availability

R code to run the models and generate Figure 4 is available on the OpenScience Framework. DOI: 10.17605/OSF.IO/QTRG9
