# Supplementary material for: Trophic interaction modifications: an empirical and theoretical framework
Source: Ecol Lett. 2017 Sep 17;20(10):1219–30. doi: 10.1111/ele.12824 (PMC6849598; doi:10.1111/ele.12824)
Supplement: Supplementary file 1 [file ELE-20-1219-s001.docx]

Appendices

Appendix 1. Functions used to describe relationship between modifier species density and functional response parameter.

| Name | Examples of Use | Function | Lower Bounded above 0? | Upper Bounded? | Contains threshold? | Number of free parameters |
| --- | --- | --- | --- | --- | --- | --- |
| Linear | Arditi *et al.* (2005) | $max\{0, 1+X_{k}c_{ijk}\}$ | N | N | N | 1 |
| Exponential | Křivan & Schmitz (2004)  Okuyama & Bolker (2012) | $exp(c_{ijk} X_{k})$ | N | N | N | 1 |
| Power Law | Goudard & Loreau (2008)  Lin & Sutherland (2014) | $\left( 1+X_{k} \right)^{c_{ijk}}$ | N | Y | N | 1 |
| Interference | Paixão *et al.* (2014) | $\frac{1}{1-c_{ijk}X_{k}}$ | N | N | N | 1 |
| Saturating | Kéfi *et al.* (2012)  Sanders *et al.* (2014) | $\frac{\exp\left( c_{ijk} \right)\times X_{k}+T_{0}}{X_{k}+T_{0}}$ | Y | Y | N | 2 |
| Logistic | Kéfi *et al.* (2012) | $\frac{M_{nti}-M_{0}}{1+e^{a\left( X_{k}-d \right)}}+M_{0}$ | Y | Y | Y | 3 |
| Dose Response | Ramos-Jiliberto *et al.* (2008) | $1+\frac{c_{ijk}\left( X_{k} \right)^{v}}{\left( X_{k} \right)^{v}+u^{v}}$ | Y  (if $c_{ijk}$ > -1) | Y | Y | 3 |

Table S1. Expressions that have been suggested or used for the modification of functional response parameters. $c_{ijk}$ = Modification Parameter (k’s effect on the consumption of i by j), $X_{k}$ = Modifier Density, $T_{0}$ = Half-Saturation Density of Modification, a = plays role in determining steepness of threshold, direction of effect and intercept, d= point of inflection, also affects intercept. $M_{nti}$= value when $X_{k}$ tends to infinity, $M_{0}$ = baseline value, v = curve abruptness, u = modifier density when μ (modification effect) = 0.5. Some models have been adapted from the original source for consistency in the table. Note that the large number of expressions for predators adaptively foraging between multiple prey that have been put forward but not in a TIM context have not been included in this table.


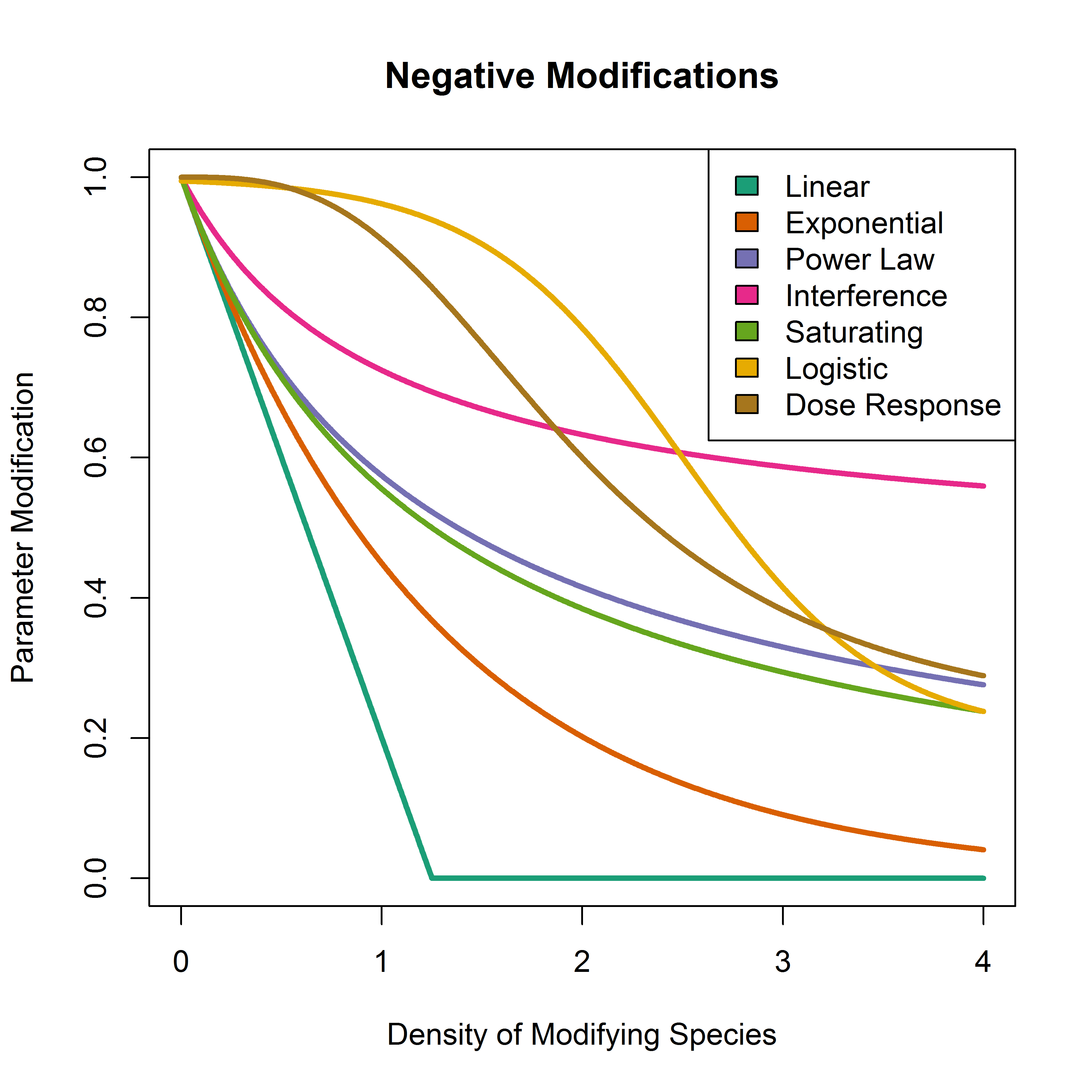

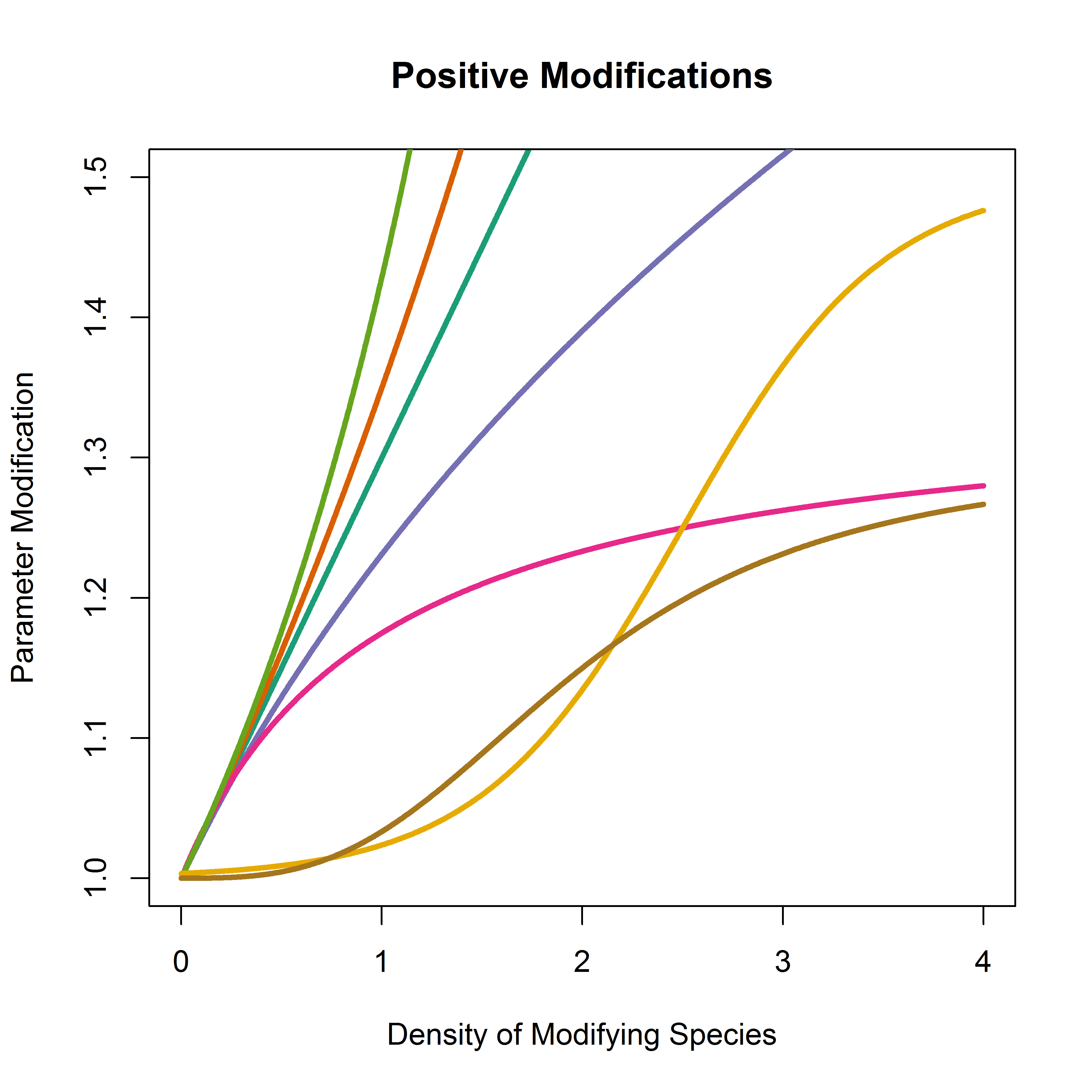


Figure S1. Graphical depictions of example modification functions in Table 1. For the negative modifications $T_{0}$ = 1${, M}_{nti}$ = 0.2, $M_{0}$= 1, d = 2.5, u=2, v=3,$c_{ijk}$= -0.8, a= -2. For positive modifications values are the same except:$c_{ijk}$ = 0.3,$M_{nti}$ = 1.5.

Appendix 2. Derivation of Relative Change in $B_{CR}$ TIM Metric

$B_{CR}$ is an interaction strength metric that represents the biomass potential of the resource that is captured by the consumer. It was originally put forward by Gilbert *et al* (2014) in their study of the effect of temperature on interaction strength given temperature affects multiple parameters simultaneously. It has been used by Nilsson & McCann (2016) to show that this metric (which they term *'New IS'*) relates in a straightforward and meaningful way to stability. It uses resource biomass accumulation and consumer relative growth to define $B_{CR}$ in terms of biomass flow through the consumer- resource system. This flow is related to static measures of the resource biomass (K/R*) and has the advantage of being interpretable both experimentally and theoretically.$K$ in this context is seen as an aggregate of supply rate and resource loss rate.

$B_{CR}$ is defined (Gilbert et al 2014, Eq 3.) where both resource and consumer are able to coexist as:

$$B_{CR}\propto\frac{[Consumption Efficency][R Biomass Accumulation]}{[ConsumerLoss]}=\frac{[ef(R)][K]}{[m(C)]}=\frac{K}{R^{*}}=\frac{R without consumer}{R with consumer}$$

$B_{CR}$ measures can be represented by aggregates of parameters. Both papers present how these values can be derived from parameter sets for different models with different functional responses. It can be used for higher trophic levels using appropriate measures of equilibrium biomasses of prey in place of K. Eg: Type I functional response (Gilbert et al 2014, Eq 5.):

$$B_{CR}\propto\frac{R^{*} without consumer}{R^{*} with consumer}=\frac{K}{m/ea}=\frac{eaK}{m}$$

In a system where a modifier species affects how a consumer affects a resource it is possible to use this measure of interaction strength in a measure of interaction strength change. Using the definition of:

$$B_{CR}\propto\frac{R without consumer}{R with consumer}$$

We suggest a metric of the strength of a TIM could be the ratio of this new metric with and without the TIM:

$$\Delta B_{CR}=\frac{B_{CR}+TIM}{B_{CR} 0 TIM}=\frac{\frac{R without consumer+TIM}{R with consumer+TIM}}{\frac{R without consumer 0 TIM}{R with consumer 0 TIM}}$$

Since the TIM would not be having an effect when there is no consumer this can be simplified twice:

$$TIM=\frac{\frac{R without consumer}{R with consumer+TIM}}{\frac{R without consumer}{R with consumer 0 TIM}}=\frac{R with consumer+TIM}{R with consumer 0 TIM}$$

This gives some stronger justification for using this otherwise quite simple metric of TIMs, the change in equilibrium density of the resource caused when the TIM is present. It has the advantage of being composed of calculable parameters.

As an example, here we calculate the change in $B_{CR}$ in a minimal three species Lotka-Volterra with logistic growth and a TIM from modifier species M denoted by $f(M)$:

$$\delta M/dt=eaMC-nM$$

$$\delta C/dt=ef(M)aCR-aMC-mC$$

$$\delta R/dt=r(1-R/K)R-f(M)aCR$$

The effect of the TIM could be defined from the non-zero equilibria, showing that in this case the strength of the TIM depends on a particular subset of parameters and the magnitude of $\Delta B_{CR}$ is not directly related to f(M) due to the 1- term.

$$C^{*}=n/ea$$

$$R^{*}=K(1-\frac{af(M)C^{*}}{r})=K(1-\frac{f(M)n}{er})$$

$$M^{*}=ef(m)R^{*}-m/a)=ef(m)K(1-\frac{f(m)n}{er})-m/a)$$

$$\Delta B_{CR}=\frac{R with consumer+TIM}{R with consumer 0 TIM}=\frac{K(1-\frac{f(M)n}{er})}{K(1-\frac{n}{er})}=\frac{1-\frac{f(M)n}{er}}{1-\frac{n}{er}}$$

Appendix 3. Measuring TIMs in cycling systems.

One approach to quantify the impact of a TIM on the dynamics of a particular system would be to measure the coefficient of variation (CV) of key values such as μ or biomass flux. This approach is not always straightforward as fluctuations in biomass flux that would be present in the system without the TIM would need to be taken into account.

As an example system, the non-linear tri-trophic system detailed below can be parameterised to give stable cycles, as indicated by the positive CV of predator density in Fig S4a when c = 0.


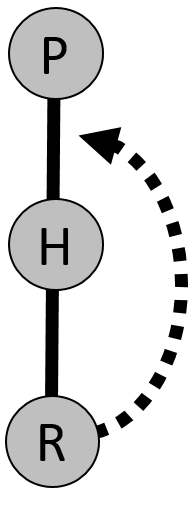

$$\frac{dP}{dt}= \frac{{\mu e_{p}a}_{p}HP}{1+\mu h_{p}a_{p}H}-m_{p}P$$

$$\frac{dH}{dt}= \frac{{e_{h}a}_{h}RH}{1+h_{h}a_{h}R}-\frac{{\mu a}_{p}HP}{1+\mu h_{p}a_{p}H} -m_{h}H$$

$$\frac{dR}{dt}= GR\left( 1-\frac{R}{k} \right)-\frac{a_{h}RH}{1+h_{h}a_{h}R}$$

$$\mu=\exp\left( c_{ijk}R \right)$$

$a_{h}$= 5, $h_{h}$ = 1.24, $m_{h}$= 0.4, $e_{h}$= 1,$a_{p}$=0.8, $h_{p}$= 2.5, $m_{p}$= 0.08, $e_{p}$= 1, $c_{\mathrm{ijk}}$ = 0, G = 1, K= 0.58

When an exponential TIM from the resource onto the predator-herbivore interaction is included, it can be seen that initially moving 𝑐𝑖𝑗𝑘 above or below 0 leads to an increase in the CV of the parameter modification (μ) (Fig S2b). With larger positive values of c, the values for CV of μ continue to rise. However, in this model a negative TIM is stabilising (Fig S2a) and past a point (marked ‘*’) the CV of μ starts to decline to 0 as the system is forced towards an equilibrium (Fig S2b). A strongly negative TIM has in this case had a strong effect on the macro-dynamics of the system, but in doing so has masked its own dynamic effect.


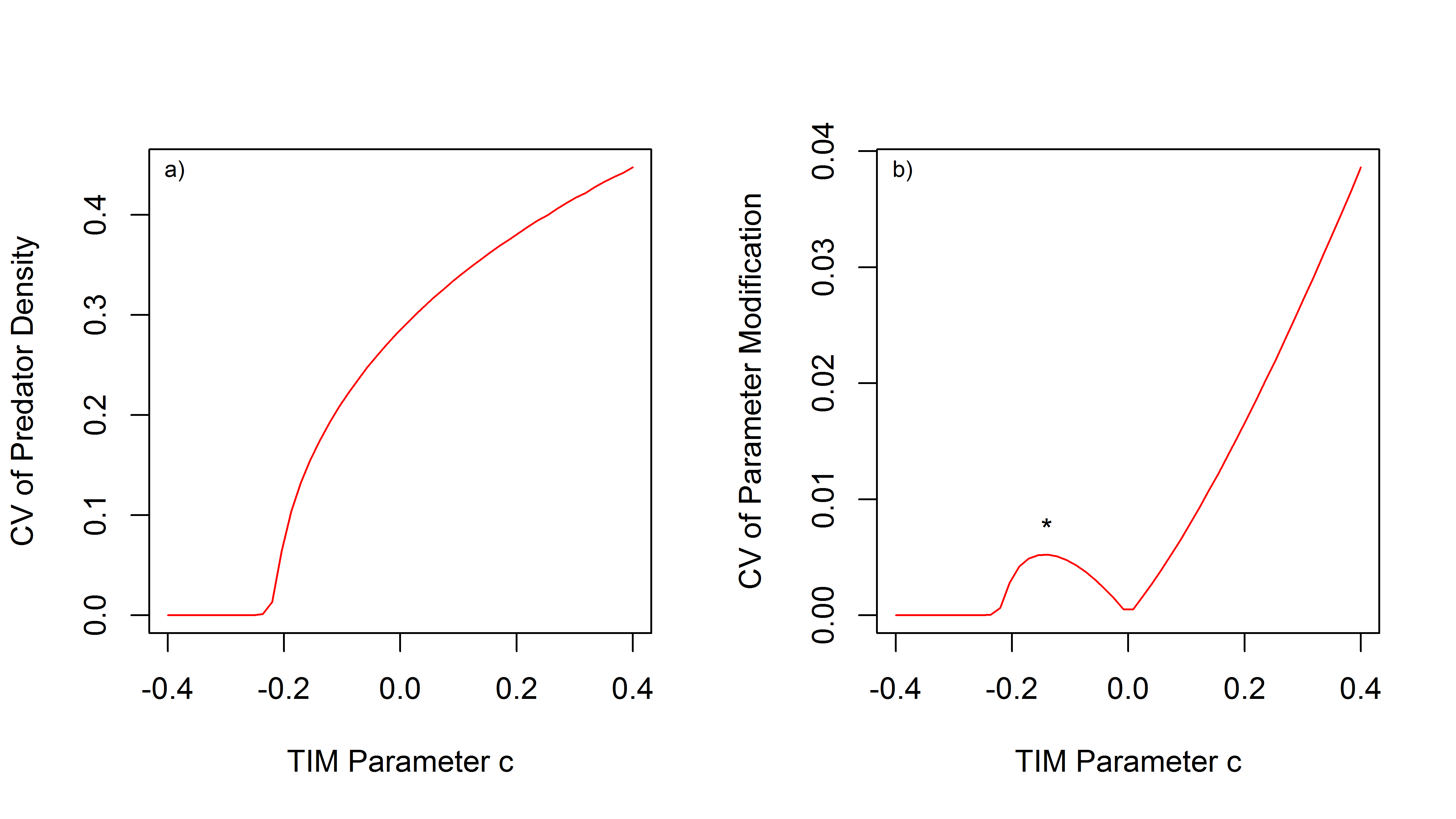


Figure S2. Plots of metrics for TIMs in a cycling system. A) coefficient of variation (CV) of the predator population density, b) CV of the parameter modification term (μ)

Appendix 4. Model details for metric comparisons.

Model structures and parameters used in the Figure 3 of the main text.

**‘Linear’ Model:**

$$\frac{dA}{dt} =a'AB- mA$$

$$\frac{dB}{dt}=bBC -a'AB- nB$$

$$\frac{dC}{dt}=rC\left( 1-\left( \frac{C}{K} \right) \right) - bBC$$

$$a'=a+cC$$

*a = 5, b = 3, r = 4, c = 0, m = 1, n = 1, K = 1*

**‘Non-linear’ Model:**

$\frac{dA}{dt}= \frac{a'AB}{1+ a^{'}hB}-mA$

$$\frac{dB}{dt}= \frac{bBC}{1+bhC}-\frac{a^{'}AB}{1+a^{'}hB} -nB$$

$\frac{dC}{dt}= rC\left( 1-\frac{C}{K} \right)-\frac{bBC}{1+bhC}$

$$a'=ae^{cR}$$

*a = 6, b = 5, r = 2, c = 0, m = 1, n = 1, h= 0.4, K = 1*

**‘Aphid-Parasitoid’ Model** of van Veen *et al* (2005):

$\frac{dA}{dt}= \frac{saAB}{1+ bB+cC+dA}-mA$

$$\frac{dB}{dt}= r_{B}B\left( 1-\alpha_{BB}B-\alpha_{BC}C \right)- \frac{aAB}{1+ bB+cC+dA}$$

$$\frac{dC}{dt}= r_{C}C\left( 1-\alpha_{CC}C-\alpha_{CB}C \right)$$

Parameters have been renamed for consistency with the other models. In order to fit the populations on the same scale, the population densities of the aphids and parasitoids are displayed on a log-scale.

A = Parasitoid wasp, (*A. ervi*), B = Host aphid (*A. pisum*), C= Competitor aphid (*M. viciae*).

*a =0.000382,* $a_{BC}$*=0.00037*$, a_{CB}$*=0.000397,* $r_{B}$ *=3.22,* $r_{C}$*=2.82,* $a_{A}$ *=0.281, s =0.5, d =1.26, b =0.0233, m =0.634.* Fitted value of *c=0.043*

Appendix 5. Comparison of TIMs with direct parameter changes

In stable systems, the introduction of a TIM can have equivalent effects to directly changing TIM parameters. As a demonstration, we examine the tri-trophic system detailed in Appendix 3 above, with a TIM introduced using an exponential link function and a standard set of parameters where the producer modifies the top predator-consumer interaction.

Stability is calculated from eigenvalues of the Jacobian at the system roots. A system with parameters that would lead to a cycling system can be stabilised either by reducing the attack rate of the predator directly (Fig S3a), or by increasing the negative effect of the TIM (Fig S3b). The stabilising effect of the TIM cannot therefore be directly attributed to the extra dynamic components of the TIM. For this an additional step is necessary, for example a close examination of the point at which the stability is attained. It is possible to infer the resultant post-modification attack rate and compare to direct modification diagram. Here the system with direct change becomes unstable at an attack rate of approximately 0.66. With the TIM the equivalent boundary between stability and instability occurs at 𝑐𝑖𝑗𝑘 = -0.267, which is equivalent in this model to a μ of 0.876 and hence a predator attack rate of 0.7. This means that this system with a resultant attack rate of 0.68 would be stable when produced by this TIM, but unstable in a system with fixed parameter values, demonstrating an additional role for the dynamic processes. Note that towards the left of the diagrams in this system there is a very strong divergence between the impact of lower fixed attack rates (which do not permit even unstable systems to exist) and large negative effects of the modification, which can lead to persistent (albeit very large) cycles.


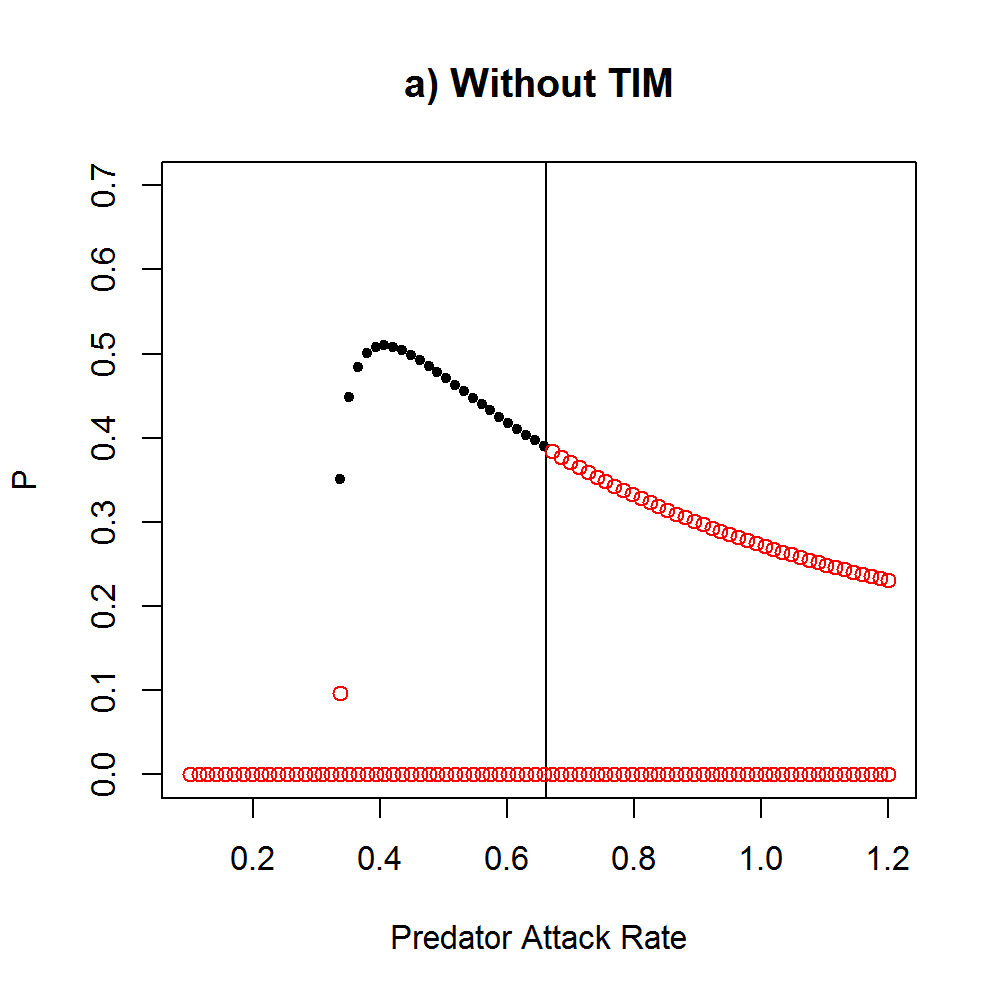

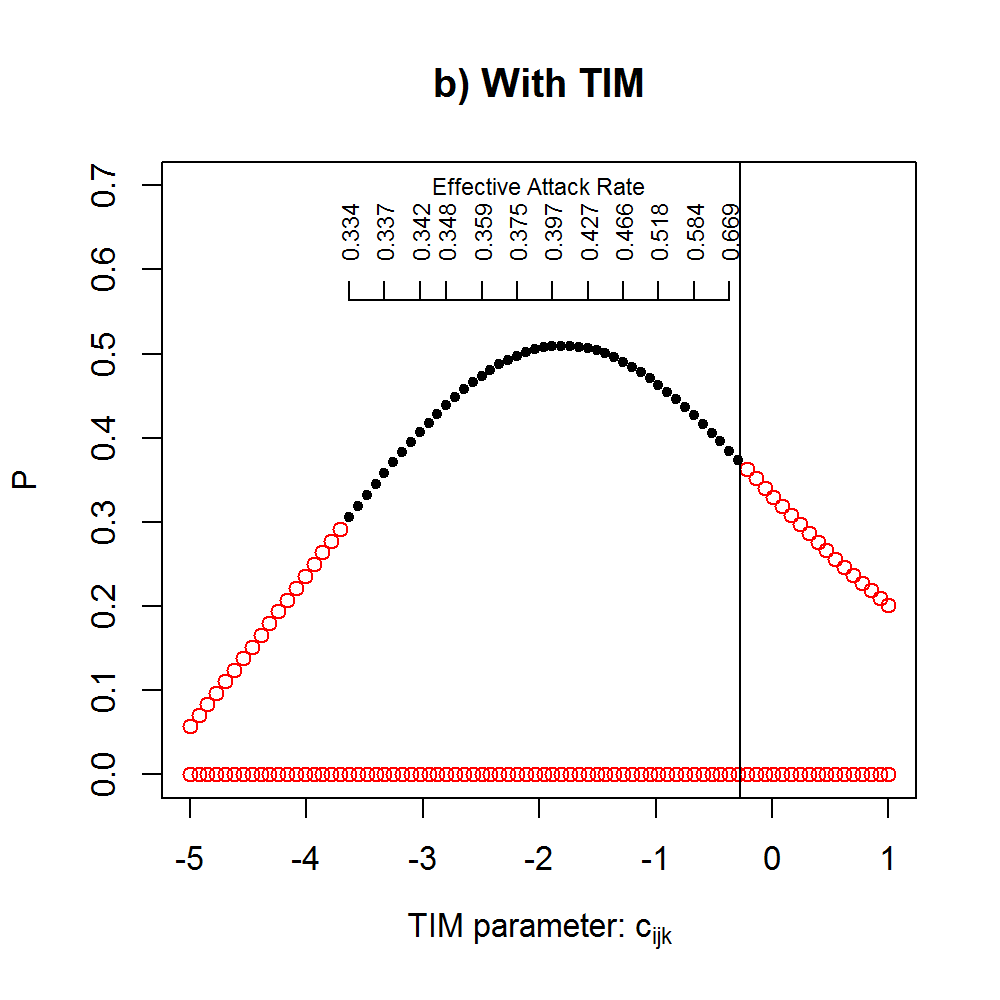


Figure S3. Stability diagrams comparing direct modification of attack rates (a) to the introduction of a TIM (b). Stable roots are shown as black dots, unstable with red circles.

**References for Supplementary Material**

Arditi, R., Michalski, J. & Hirzel, A.H. (2005). Rheagogies: Modelling non-trophic effects in food webs. *Ecol. Complex.*, 2, 249–258

Gilbert, B., Tunney, T.D., McCann, K.S., DeLong, J.P., Vasseur, D.A., Savage, V., *et al.* (2014). A bioenergetic framework for the temperature dependence of trophic interactions. *Ecol. Lett.*, 17, 902–914

Goudard, A. & Loreau, M. (2008). Nontrophic Interactions, Biodiversity, and Ecosystem Functioning: An Interaction Web Model. *Am. Nat.*, 171, 91–106

Kéfi, S., Berlow, E.L., Wieters, E.A., Navarrete, S.A., Petchey, O.L., Wood, S.A., *et al.* (2012). More than a meal… integrating non-feeding interactions into food webs. *Ecol. Lett.*, 15, 291–300

Křivan, V. & Schmitz, O.J. (2004). Trait and density mediated indirect interactions in simple food webs. *Oikos*, 107, 239–250

Lin, Y. & Sutherland, W.J. (2014). Interaction modification effects on ecological networks are affected by ratio dependence and network topology. *J. Theor. Biol.*, 363, 151–157

Nilsson, K.A. & McCann, K.S. (2016). Interaction strength revisited—clarifying the role of energy flux for food web stability. *Theor. Ecol.*, 9, 59–71

Okuyama, T. & Bolker, B.M. (2012). Model-based, response-surface approaches to quantifying indirect interactions. In: *Trait-Mediated Indirect Interactions: Ecological and Evolutionary Perspectives* (eds. Ohgushi, T., Schmitz, O. & Holt, R.D.). Cambridge University Press, Cambridge, pp. 186–204

Paixão, E.A., Costa, M.I. da S. & Faria, L.D.B. (2014). The influence of the trade-off between consumer-foraging and predation risk on tritrophic food chain dynamics. *Ecol. Complex.*, 20, 201–207

Ramos-Jiliberto, R., Mena-Lorca, J., Flores, J.D. & Morales-Álvarez, W. (2008). Role of inducible defenses in the stability of a tritrophic system. *Ecol. Complex.*, 5, 183–192

Sanders, D., Jones, C.G., Thébault, E., Bouma, T.J., Heide, T. Van Der, Belzen, J. Van, *et al.* (2014). Integrating ecosystem engineering and food webs. *Oikos*, 123, 513–524

van Veen, F.J.F., van Holland, P.D. & Godfray, H.C.J. (2005). Stable Coexistence in Insect Communities Due to Density- and Trait-Mediated Indirect Effects. *Ecology*, 86, 3182–3189

Vos, M., Kooi, B.W., DeAngelis, D.L. & Mooij, W.M. (2004). Inducible defences and the paradox of enrichment. *Oikos*, 105, 471–480
